# Weighting by heritability for detection of quantitative trait loci with microarray estimates of gene expression

**DOI:** 10.1186/gb-2005-6-3-r27

**Published:** 2005-02-28

**Authors:** Kenneth F Manly, Jintao Wang, Robert W Williams

**Affiliations:** 1 Department of Pathology, University of Tennessee Health Science Center, 855 Monroe Avenue, Memphis, TN 38163, USA; 2 Department of Anatomy and Neurobiology, Center of Excellence in Genomics and Bioinformatics, University of Tennessee Health Science Center, 855 Monroe Avenue, Memphis, TN 38163, USA; 3 Department of Biostatistics, 246 Farber Hall, University at Buffalo, Buffalo, NY 14214, USA

## Abstract

Heritable differences in transcribed RNA levels can be mapped as quantitative trait loci (QTLs). Transcribed RNA levels are often measured by hybridization to microarrays of oligonucleotide probes, in which each transcript is represented by multiple probes. The use of recombinant inbred lines allows an estimate of the heritability of expression measured by individual probes. This heritability varies greatly. We have tested heritability-weighted averages to define expression of a transcript and found that these allow detection of more QTLs than previously described methods.

## Abstract 2

The use of recombinant inbred lines allows an estimate of the heritability of expression measured by individual probes. By testing heritability-weighted averages to define expression of a transcript, more QTLs can be detected than with previously described methods.

## Background

The steady-state abundance of an RNA species in an organ is, in part, genetically controlled and can be considered a quantitative genetic trait. Microarray methods for estimating RNA sequence abundance [1], combined with genetic methods for identifying loci affecting quantitative traits [2-4], provide the opportunity to survey tissues for all genetically controlled variation in gene expression. This approach has been called genetical genomics [5], and its feasibility has been demonstrated in experimental crosses and human populations [6-10].

Genetical genomics is further enhanced by using recombinant inbred lines as a mapping population. The use of recombinant inbred lines allows comparison of gene expression among different tissues and the comparison of gene expression with classical physiological and behavioral traits from the published literature [11,12]. Public datasets and online software at WebQTL [10,13] allow free exploration of the characteristics of this form of analysis [14]. In addition, recombinant inbred lines can provide both replicates from genetically identical individuals and samples from different segregants. Data from these define genetic and non-genetic variation, define a measure of heritability for expression of individual genes, and provide the basis for a new method of data reduction for genetical genomics.

Data reduction is an issue because Affymetrix GeneChip oligonucleotide microarrays assay each target mRNA with a set of 11 to 16 pairs of 25-nucleotide DNA probes. Each pair of probes consists of a perfect match (PM) sequence and a mismatch (MM) sequence, the latter intended to estimate nonspecific binding. The Affymetrix software Microarray Suite 4.0 and 5.0 (MAS 4 and MAS 5) estimate expression from the average difference of PM and MM fluorescence. Since the pioneering study of Li and Wong [15], however, it has been clear that MM binding includes target-specific binding as well as nonspecific binding, and the appropriate use of MM fluorescence has been an open question. In fact, a recent publication shows that it may be more useful to use the sum of PM and MM values instead of their difference [16]. In short, the behavior of oligonucleotide microarrays is not adequately explained by models that only consider base complementarity. More realistic models consider nonspecific binding, saturation, the effects of fluorescent labeling and intramolecular folding of target and probe [15,17-19].

Several alternative methods have been proposed to combine multiple probe-specific values into a single expression estimate. Three widely used alternatives are robust multiarray average (RMA) [20], model-based expression index/intensity (MBEI), implemented in dChip software [15], and positional-dependent nearest-neighbor model (PDNN) [17]. RMA provides statistically robust averaging methods, dChip fits a model that allows probe-specific binding affinities, and PDNN fits a model that allows sequence-specific binding affinities and nearest-neighbor stacking interactions. A weighted-average method is also available, one which weights probe-specific values by a cross-validation procedure [21]; this method, however, does not take advantage of replicate microarrays and the current implementation in Bioconductor [22] is too slow for this application. Finally, a method (SUM) based on the sum of PM and MM values has recently been described [16]. The rationale for this method is that MM probes exhibit probe-specific binding as well as nonspecific binding [15,17] and may therefore be more effective for estimating specific binding than for correcting for nonspecific binding. Indeed, the SUM method outperforms MAS5 in several respects.

We describe here a new method, specifically designed for application to genetical genomics. In this method, called heritability-weighted transform version 1 (HWT1), probe-specific data is combined in a weighted average in which the weights are determined by an estimate of the heritability of the data for each probe.

## Results

**Figure 1:**
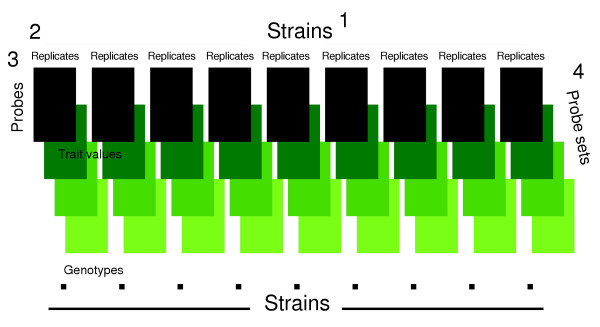
The four-dimensional nature of microarray data used for QTL mapping. Recombinant inbred lines (strains) comprise dimension 1; the replicate arrays for each strain, dimension 2. Multiple probes for each probe set comprise dimension 3, and multiple probe sets (transcripts), dimension 4. Green rectangles represent the multiple probe- and replicate-specific expression values that must be collapsed to a single value for QTL mapping. That mapping correlates expression values with genotypes in dimension 1. Heritability-weighted averaging uses information in dimensions 1 and 2 to collapse dimension 3 by weighted averaging. Dimension 2 is collapsed by unweighted averaging.

Figure 1 provides an overview of the dataset and the data reduction problem for QTL mapping with gene-expression data from recombinant inbred strains. These gene-expression data form a four-dimensional dataset. As shown in Figure 1, the first dimension is formed by recombinant inbred strains; the second by replicate samples from each strain; the third by multiple probes of each probe set; and the fourth by multiple probe sets representing different transcripts. For QTL mapping, dimensions 2 and 3 must be collapsed to single values that can be compared with genotypes for each strain (in dimension 1). Normally, dimensions 2 and 3 are collapsed by simple averaging or by averaging probe differences.

Heritability is determined by the relative expression variance contributed by dimensions 1 and 2. The HWT1 method described here uses this information from dimensions 1 and 2 to define weights that allow dimension 3 to be collapsed with a weighted average. Dimension 2 is still collapsed with a simple average.

**Figure 2:**
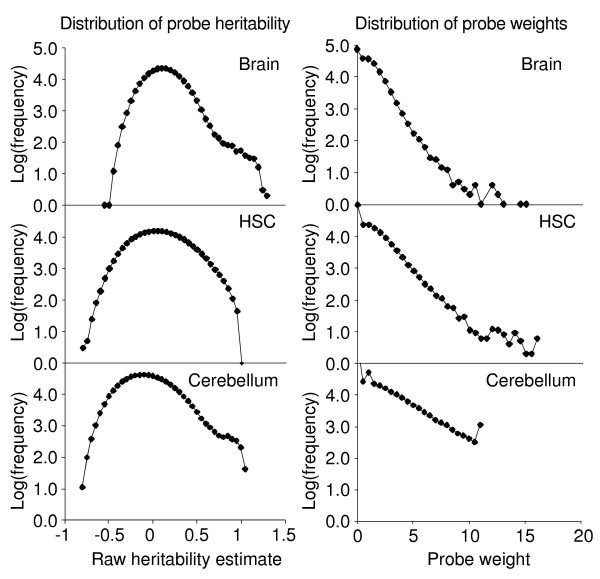
Distribution of heritability of probe intensities and of probe-specific weights derived from heritability. Frequencies are shown on a log scale to make the tails of the distributions visible. Expression is in BXD RI lines from the tissue indicated; HSC, hematopoietic stem cells. The left-hand panels show the distribution of raw heritability estimates for individual Affymetrix probes. The right-hand panels show the distribution of probe-specific weights derived from those heritability estimates.

The left-hand panels of Figure 2 show the distribution of estimated heritability of expression for individual PM probes, with frequencies shown on a log scale to make the tails of the distribution visible. Results from three organs or tissues from BXD recombinant inbred lines are shown: brain expression (Brn); hematopoietic stem cell expression (HSC); and cerebellum expression (Cer). Brain and HSC were assayed with Affymetrix U74Av2 microarrays; cerebellum with Affymetrix M430A and B. In all datasets, estimates range from well below 0 to 1 or slightly above. The method used for estimating heritability is known to yield estimates outside the natural range expected for heritability [23]. Indeed, as shown in Figure 2, 21%, 45% and 60% of estimates are negative (for brain, HSC and cerebellum, respectively) and a few (< 0.1%) of brain and cerebellum estimates are above 1.0.

Although estimation methods exist that would avoid these values, the current method is simple and serves the intended purpose if negative heritabilities and those above 1 are adjusted by assigning them values of 0 and 1, respectively. When these adjusted heritabilities are normalized by the average (adjusted) heritability of probes in each probe set, the resulting weights are distributed as shown in the right-hand panels of Figure 2. About 36%, 49%, and 61% (for brain, HSC, and cerebellum, respectively) of probe weights are zero and 55%, 60%, and 66% are less than 1.0. These probes are fully or partly excluded from any weighted average. A small minority of probes, less than 3%, receive weights above half the maximum possible weight, suggesting that they will dominate the average for the probe set to which they belong.

**Figure 3:**
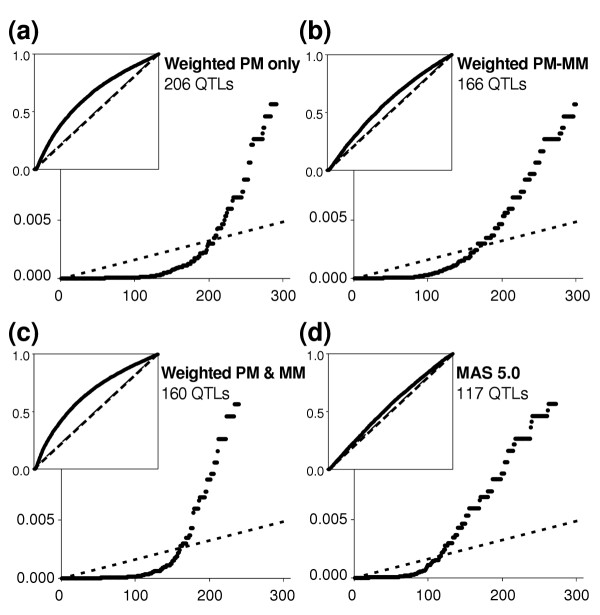
Distribution of P-values from QTL mapping of brain RNA expression. The P-value for the best QTL for each microarray probe set is plotted against the rank of that P-value among all probe sets [24]. The four figures show four methods of pre-processing the data before QTL mapping. In each panel the smaller inset shows the entire range of P-values and the larger figure shows the smallest 200 to 300 P-values. The dashed line in the inset shows the expected distribution for random P-values; in the larger figure the dashed line shows the limit for 20% false-discovery rate, according to Benjamini and Hochberg [25]. **(a) **HWT1 weighting of PM values only; **(b) **HWT1 weighting of PM-MM differences; **(c) **HWT1 weighting of PM and MM values combined; **(d) **PM-MM differences averaged by Affymetrix MAS 5.0 software.

The results of QTL mapping with weighted averages are shown in Figure 3, in which sorted P-values from a set of microarrays is plotted against the rank of each P-value [24]. Each P-value represents the significance of the best single QTL, that is, of the best association between expression of one transcript and genotypes at some marker. In this plot, uniformly distributed P-values, from tests in which the null hypothesis is always true, form a straight line along the diagonal. That is, a complete absence of QTLs would yield a straight diagonal line. In each panel, an inset shows the entire range of P-values, most of which do approximately form a diagonal. The main figure shows the smallest values only. In each main figure the line formed by the P-values bends sharply, indicating a local excess of small P-values. Those P-values which fall below the dotted line in each panel form a group in which the false-discovery rate is expected to be no greater than 20%, according to a Benjamini and Hochberg test [25]. This criterion is used throughout this paper to define significant QTLs.

The panels of Figure 3 compare QTLs detected after averaging with Affymetrix MAS 5.0 software and QTLs detected with three variations of heritability-weighted averaging. These variations differ in their use of MM probes. As transcript binding to MM probes seems to include both nonspecific and target-specific binding [15,17,18], we tested both subtracting MM values from PM (to remove nonspecific signal) and adding MM values to PM (to add target-specific signal). Figure 3a shows results obtained by calculating heritability from PM probes and averaging only those probes, Figure 3b shows results obtained by calculating heritability from and averaging PM - MM differences and Figure 3c shows results obtained by calculating heritability from and averaging all probes (PM and MM) together. Using 20% false-discovery rate as a significance cutoff, each of the heritability-weighting methods yields more QTLs than MAS 5.0. With this dataset, using only PM probes yielded more QTLs than the other two weighting methods.

When weighted expression averages were randomly permuted among the recombinant inbred (RI) strains before mapping, no QTLs were detected at 20% false-discovery rate (data not shown). Since heritability estimates are unaffected by permutation, permuting data after weighted averaging is equivalent to permuting before averaging. Furthermore, simulation showed that heritable variation alone is not sufficient to define QTLs. Simulated datasets were generated with heritable variation distributed among probes in various ways, including one in which all heritable variation was generated for a single probe of each probe set. In these simulated datasets all variation was independent of marker genotypes. No QTLs were detected from these simulated datasets after heritability-weighting and QTL mapping (data not shown).

There is little relationship between the abundance of transcripts and the likelihood of detecting a QTL (data not shown). If anything, strong QTLs tend to be found among transcripts of moderate abundance. This tendency might be explained if apparent interstrain variation, necessary for QTL detection, is reduced when abundance is extreme, either near the lower limit of detection or high enough to saturate some oligonucleotide probes.

**Figure 4:**
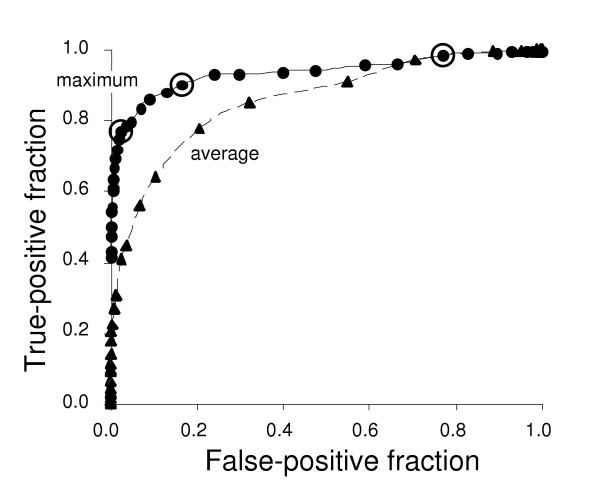
Probe heritability as a predictor of a detectable QTL for a probe set. The figure shows receiver operating characteristic (ROC) curves for prediction of existence of a detectable QTL by either average heritability or maximum heritability among probe-specific data in a probe set. The true-positive fraction on the ordinate is the fraction of probe sets with a significant QTL that are identified as such by selection at a given maximum heritability. The false-positive fraction is the fraction of probe sets without a significant QTL that are selected as having a QTL at the same maximum heritability. Triangle symbols show ROC curve for average heritability; circle symbols show ROC curve for maximum heritability. Circled points are explained in the text

Probe heritability is a predictor of the existence of a detectable QTL for a probe set. Either average heritability or maximum heritability among probes in a probe set can be used as a predictor. In either case, heritability above a threshold value is taken to predict the existence of a QTL. Figure 4 shows the receiver operating characteristic (ROC) curves for average or maximum probe heritability used as a predictor of the existence of a significant QTL. The ordinate shows the fraction of transcripts with QTLs that are correctly predicted as such by heritability; the abscissa shows the fraction of transcripts without QTLs that are incorrectly predicted by heritability to have a QTL. The curves are produced by plotting these two quantities for various threshold values for average heritability or maximum heritability. For a perfect predictor, the ROC curve would follow the left and top boundaries of the figure. For a useless predictor, the ROC curve would be a diagonal line between the origin and the upper-right corner.

These curves show that maximum heritability is more effective than average in predicting a detectable QTL. Because probe sets that do not define a significant QTL greatly outnumber those that do, probe sets defining a QTL are still a minority among probe sets selected for heritability. This situation is illustrated by three points that are circled in the figure. The right-hand circled point shows that selecting for maximum heritability greater than 0.35 selected 77% of probe sets; 2% of these yielded QTLs composing 99% of all QTLs. The center circled point shows that a threshold of 0.525 selected 17% of probe sets, of which 8% yielded QTLs composing 90% of QTLs. The left-hand circled point shows that a threshold of 0.675 selected 4% of probe sets, of which 32% yielded QTLs composing 75% of QTLs.

**Figure 5:**
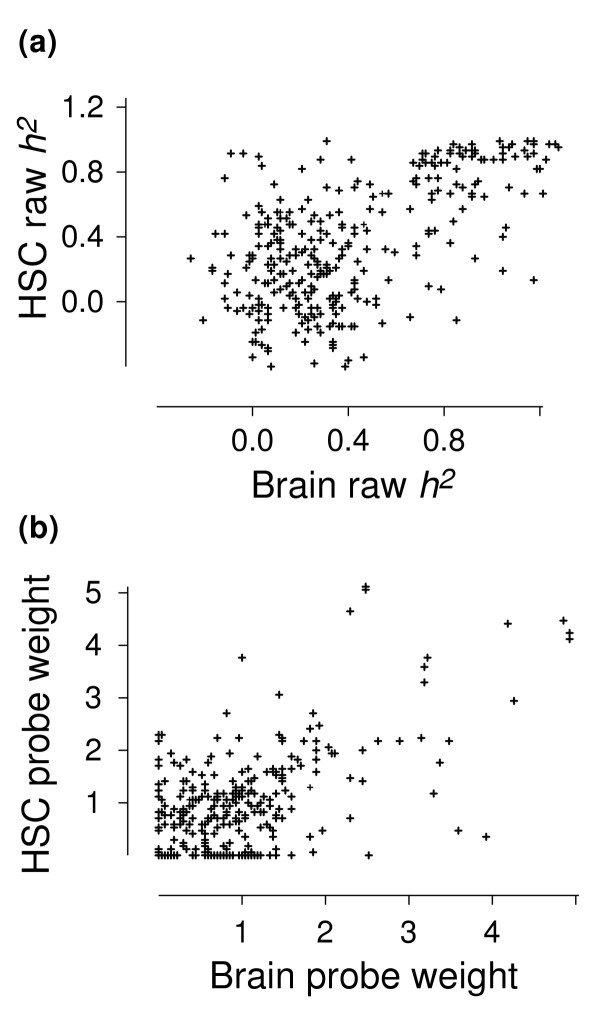
Poor correlation of heritability and heritability-derived weights. The figures compare the raw heritability (*h*^2^) and weights of probes from probe sets in which at least one probe had a raw heritability greater than 0.90 in both brain and HSC data. **(a) **Raw HSC heritability for each probe vs raw brain heritability; **(b) **probe weight for HSC data vs weight for brain data.

The availability of RNA from unrelated tissues, brain and HSC, allowed us to consider the question of whether probe heritabilities are specific to the tissue of origin. Raw probe heritabilities for data from brain and HSC have a correlation coefficient of -0.004, but that value means little because most probe heritabilities are close to zero. A more meaningful comparison is between probe heritabilities for probe sets in which at least one probe has significant heritability. Figure 5 shows scatterplots comparing brain and HSC raw probe heritability and probe weight for 304 PM probes (19 probe sets) in which at least one probe from each organ had heritability greater than 0.90. Even with this degree of selection, the correlation for heritability or weight is only 0.59 or 0.58, respectively. Thus, even with extreme selection, there is little correlation between probe heritabilities from these two sources, suggesting the probe heritabilities are tissue specific.

QTLs for gene expression can be classified according to the chromosomal location of the QTL relative to the location of the gene being expressed. Those for which the location of the QTL and gene are tightly linked are characterized as *cis *QTLs; those for which the locations are different are *trans*. In this study the location of a QTL is defined by the location of the marker achieving the highest likelihood ratio statistic (LRS), a marker defined by a simple-sequence repeat whose location is known in the mouse sequence. *Cis *QTLs are, somewhat arbitrarily, defined as those for which this marker is within 10 megabases (Mb) of the location of the probe sequence by which the gene expression is measured.

QTLs can also be classified according to the direction of the effect on gene expression. We adopt the convention that QTLs are labeled '+' if the DBA/2J allele is associated with higher apparent expression and '-' if the C57BL/6J allele is associated higher apparent expression. Assuming that Affymetrix probe sequences were largely designed for the C57BL/6 sequence, sequence differences between C57BL/6 and DBA/2 in the sequence recognized by a probe will tend to make DBA/2 hybridize more poorly than C57BL/6. That is, variation in sequences complementary to probe sequences can create artifactual QTLs, reflecting a difference in hybridization rather than a difference in expression. Such artifactual QTLs would be expected to be *cis *-.

**Figure 6:**
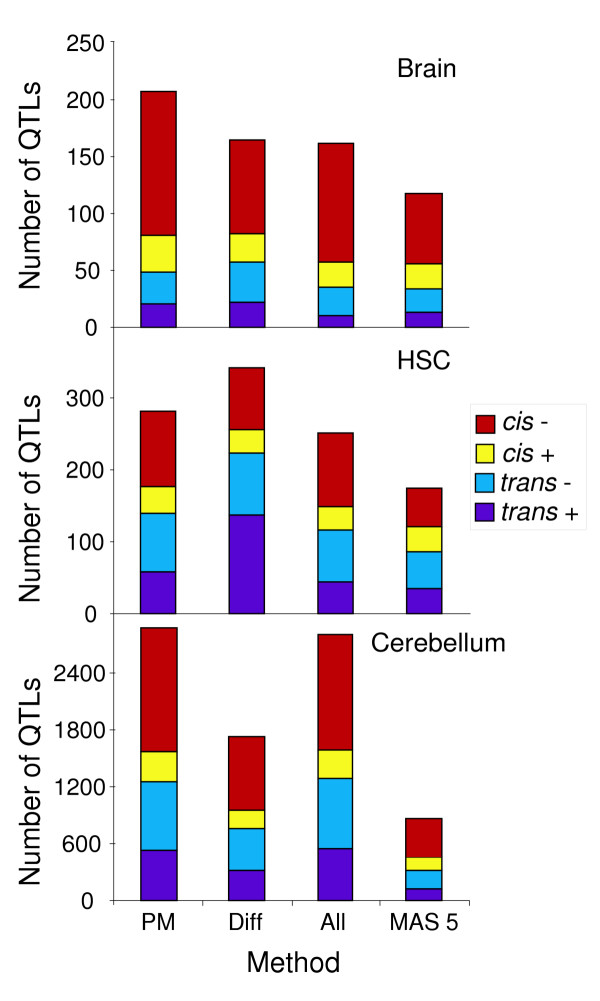
Number and types of QTLs in the three tissues defined by four methods of data summary. PM, HWT1 weighting of PM values only; Diff, HWT1 weighting of PM-MM differences; All, HWT1 weighting of PM and MM values combined; MAS 5, PM-MM differences averaged by Affymetrix MAS 5.0 software. *cis*, QTL location within 10 Mb of transcript location; *trans*, QTL location further than 10 Mb from transcript location; -, B57BL/6 allele associated with higher expression signal; +, DBA/2 allele associated with higher expression signal.

Figure 6 summarizes classification of QTLs detected by heritability-weighting methods. The three panels of the figure show data from brain, HSC and cerebellum. Each dataset confirms previous results that each of the heritability-weighted methods detects more QTLs than MAS 5.0. However, the HSC dataset differs from the other two in that weighted PM - MM differences detected more QTLs than PM probes alone.

For all methods in all datasets, *cis *- QTLs outnumber *cis *+ QTLs, in some cases by two- or threefold. This excess could be explained by polymorphisms in sequences targeted by Affymetrix probes, polymorphisms reducing the hybridization of DBA/2J RNA. For Brn and HSC the weighting procedure made some attempt to reduce this type of artifact by assigning a weight of 0 to 614 probes having known single-nucleotide polymorphisms (SNPs) in the probe target sequence. The excess of *cis *- QTLs remaining in Brn and HSC in spite of this procedure suggests that there may be additional effects from polymorphisms not included in our list.

The cerebellum dataset yielded a large number of significant QTLs. In part this yield was expected because the number of probe sets for M430 microarrays is 3.6-fold larger than for U74Av2. However, the QTL yield for the cerebellum data is about 10-fold higher than for brain or HSC, or about 2.7-fold higher relative to the number of genes represented on the microarrays. As discussed further below, the cerebellum data were obtained in two unbalanced batches, and a difference between these batches might create artifactual QTLs on chromosome 2. However, although 475 significant QTLs, 16% of the total, appear on chromosome 2, this number is too small to fully explain the large number of the cerebellum QTLs.

**Figure 7:**
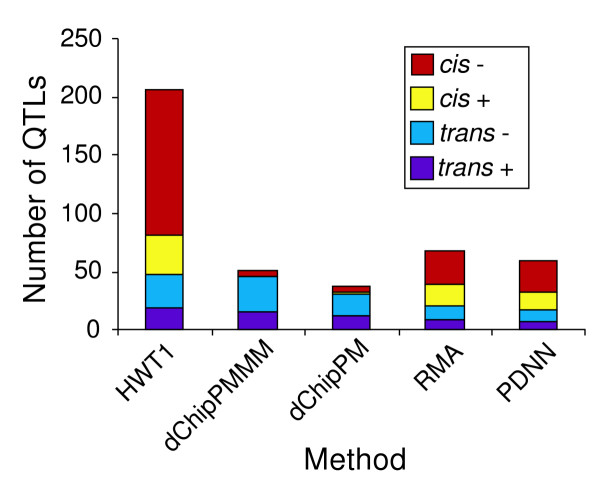
Number and types of QTLs defined in the brain dataset according to the method of data summary. Methods used were: HWT1, heritability-weighting using only PM probe data; dChipPMMM, dChip method using PM and MM probe data [15]; dChipPM, dChip method using only PM data; RMA, robust multiarray averaging using only PM probe data [20]; PDNN, PDNN method using PM and MM probe data [17]. See legend to Figure 6 for definitions of QTL types.

Figure 7 shows that the HWT1 method using only PM probes allowed the detection of more QTLs than the dChip, RMA, or PDNN data reduction methods. Compared with these methods, HWT1 detected larger numbers of QTLs in all QTL classes, but the increase in *cis *- QTLs was disproportionately large. As explained, many of those *cis *- QTLs could be artifacts caused by polymorphisms.

The number of probes that contribute to weighted averages varies considerably between probe sets. The effective number of probes can be defined, as described in Materials and methods, by a measure which is the reciprocal of a weighted average of the weights. The measure varies from 1.0, if all weights but one are zero, to the number of probes (usually 11.0 or 16.0), if all probes are weighted equally.

**Figure 8:**
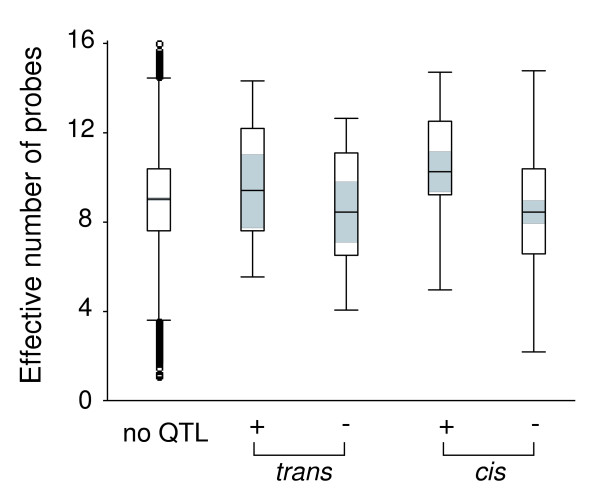
Distribution of effective number of probes in heritability-weighted averages. Boxplots show the distribution for probe sets that do not define significant QTLs (QTLs at 20% false-discovery rate) and for those that define QTLs of different types. In each plot, the central box shows the range between the 25th and 75th percentiles. The line across the box gives the median location, and the shaded area gives the 95% confidence interval for the median. Lines above and below the box give the range for all data except outliers, which are plotted singly beyond the range defined by the terminal crossbars. *trans *QTLs are those for which the QTL is more than 10 Mb distant from the location of the transcript whose expression defines it. + QTLs are those for which the DBA2/J allele is associated with higher expression.

Figure 8 shows, in boxplot form, the distribution of effective probe number for weighted averages of brain data. Five classes of probe sets are compared, those that do not define QTLs and those that define *cis *-, *cis *+, *trans *-, and *trans *+ QTLs. In each plot, the central box shows the range between the 25th and 75th percentiles. The line across the box gives the median location, and the shaded area gives the 95% confidence interval for the median.

The data in Figure 8 allow three conclusions. First, a substantial fraction of probes contribute to weighted averages that define QTLs. In each case, the central half of QTLs falls into the 7- to 13-probe interval. Although the groups do not differ significantly, there is a possible tendency for + QTLs to involve more probes than - QTLs. Finally, only the *cis *- group includes QTLs defined by fewer than four probes. QTLs that depend on so few probes are most likely to be artifactual QTLs caused by polymorphisms in the probe target sequences.

## Discussion

The heritability-weighted averaging method described here successfully summarizes oligonucleotide microarray measurements of gene expression in a way that facilitates detection of QTLs affecting that expression. It is a heuristic method, one that is not derived from an explicit statistical model. Nevertheless, the rationale is simple and rests on three facts: first, heritable variation is necessary (but not sufficient) to define a QTL; second, probes within a probe set differ greatly in the heritability of their expression estimates; and third, probes within a probe set differ greatly in their ability to detect a QTL. These facts suggested that a simple weighted average would summarize probe set data without obscuring the signal of those probes which could detect a QTL.

HWT1 is designed specifically for QTL mapping. In its present form, it does not apply to the more common experimental situation designed to estimate expression differences between samples. In that experimental situation, this method would be circular, weighting probes according to an estimate of the quantity to be estimated. QTL mapping, in contrast, does not depend directly on the differences between samples, but on the correlation of those differences with a genetic marker. Indeed, the data of Figure 4 imply the existence of a few probes with high heritability that nevertheless yield no significant QTL.

Although we designed this weighting to reflect heritability, it may, depending on the experimental design, involve more than heritability. The heritability estimate is based on the variance between strains (which includes genetically determined variance) and the variance within strains, as an estimate of non-genetic variance. This estimate is closely related to other size-of-effect measures, such as repeatability, ω^2^, η^2^, or ε^2 ^[26-29]. Although we have not tested weighting with these alternative measures, we expect any of them would provide a similar benefit for QTL mapping. However, the optimum weighting for this application is not yet determined.

The frequencies of *cis *QTLs detected in this study (31-77%) fall within the wide range of frequencies detected in other studies. The most closely comparable study is that of mouse liver transcription, in which the frequency of *cis *QTLs varied from 34% for moderately significant QTLs (log odds score (LOD) > 4.3) to 71% for more significant QTLs (LOD > 7.1) [8]. However those results were based on microarrays of 60-nucleotide probes, which would be expected to be less sensitive than Affymetrix probes to the effects of single-nucleotide polymorphisms. The same study reported a frequency of 80% for the more significant QTLs (LOD > 7.0) for maize leaves. For yeast transcription assayed with cDNA arrays, Brem and co-workers estimated 36% *cis *QTLs [7], and for a human cell line assayed with Affymetrix arrays Morley and co-workers reported 18% [9].

Variance within strains usually includes non-genetic biological variation, but that was not true for the HSC dataset, for which replicates were derived from a single biological sample. In that dataset, heritability estimates were presumably higher than if replicates had been derived from separate biological samples. Nevertheless, HWT1 weighting was clearly useful for detecting QTLs in this set.

Systematic differences among strains can affect weighting in either of two ways. Batch effects that are balanced within strains (partly true in the cerebellum data) will contribute to the within-strain variance and will deflate heritability estimates. This effect may explain why cerebellum raw probe weights include many more negative values than do brain or HSC (Figure 2). On the other hand, systematic non-genetic differences between strains (such as the batch effect in HSC data) will inflate heritability estimates. For heritability estimates, the HSC batch effect was avoided by using data from one batch.

Such batch effects may also affect QTL mapping, causing a higher frequency of false positives in areas of the genome where a batch effect fortuitously correlates with marker alleles. In fact, if the batch number in cerebellum is treated as a trait, it associates with three areas on chromosome 2 (none of which, however, reaches a suggestive level of significance). These effects could be controlled by using batch as a cofactor, both in the analysis of variance that estimates heritability and in the subsequent QTL mapping. However, these refinements go beyond what is needed to introduce the HWT1 method. Thus, in the cerebellum dataset, QTLs mapping to chromosome 2 may include false positives caused by a difference in microarray processing batch. This batch effect, however, cannot explain the exceptional number of QTLs detected in the cerebellum dataset. The excess number of QTLs detected for cerebellum (compared with brain or HSC) greatly exceeds the total number of QTLs on chromosome 2.

The comparison of heritability-weighting with other data reduction methods (Figure 7) should be considered as preliminary because they are based on results from only one set of data. More important, that comparison does not imply anything about their suitability for other purposes. In addition, modifications of any of those methods might make them more suitable for QTL mapping.

It is not clear why probes of a single probe set should vary so greatly in the heritability of their expression estimates. We suggest three possibilities. First, changes in RNA concentration will result in greatest changes in fluorescence if RNA concentrations are close to the effective binding constant for a probe. Since effective binding constants of probes vary [17-19], sensitivity to changes will vary. Second, nonspecific hybridization of probes with RNA species that do not vary among strains will reduce specific hybridization that might define a QTL. If probes differ in nonspecific hybridization, they will differ in their ability to define a QTL. Third, since probes assay different parts of the target transcript, alternative splicing and differential degradation will affect probes differently.

The QTLs described in this report were detected by fitting a single-QTL model, a statistical model assuming that all QTLs contribute to a trait with independent effects. This model can be misleading if linked and/or interacting QTLs contribute to a trait. Nevertheless, since many traits are largely controlled by one QTL or few unlinked QTLs, these results are reliable and useful. They further suggest that it may be fruitful to adapt the principle of heritability-weighting to QTL searches with multi-QTL models.

## Conclusion

To summarize expression data for individual transcripts, the HWT1 method combines probe-specific data in a weighted average in which weights are determined by the heritability of the probe-specific data. It provides a useful way to summarize datasets for genetical genomics because it places weight on probe-specific data having variation that could define a quantitative trait locus.

## Materials and methods

### Brain RNA

Brain RNA was obtained from 32 strains of BXD recombinant inbred mice, the parental strains C57BL/6J and DBA/2J, and (C57BL/6 × DBA/2)F1 hybrid. Data from parental and F1 animals were included in the heritability estimates but were not used for QTL mapping. Each individual array experiment used a pool of brain tissue (forebrain plus the midbrain, but without the olfactory bulb) that was taken from three adult animals usually of the same age. More detailed information is available at WebQTL [10]. All results derive from the 100-array December 2003 data freeze.

### Hematopoietic stem cell (HSC) RNA

Bone marrow cells were stained with lineage-specific antibodies and purified by flow cytometry. A stem-cell population was defined as the 5% cells showing least lineage-specific fluorescence [30]. Replicate samples of RNA were separately amplified from a single cell preparation for each BXD strain, and these samples were processed in two batches of 22 and eight strains. These data are described at WebQTL [10] as the March 2004 data freeze.

### Cerebellum RNA

Each individual microarray assay used Affymetrix MOE 430A and MOE430B GeneChip pairs to assay RNA from a pool of intact whole cerebella taken from three adult animals usually of the same age. RNA samples were processed in two large batches. The first batch consisted of single samples from 17 BXD strains. The second batch consisted of biological replicates for 10 strains, additional technical replicates for two strains, single samples for four additional strains, and duplicate samples for five additional strains. RNA was extracted at the University of Tennessee Health Science Center and all samples were processed at the Hartwell Center (St. Jude Children's Research Hospital, Memphis). These data are described at WebQTL [10] as the SJUT Cerebellum January 2004 data freeze.

### Microarrays

Brain and HSC data were obtained from Affymetrix U74Av2 microarrays, which provide more than 12,000 probe sets, almost all of which are represented by 16 PM probes and 16 MM probes. The cerebellum data were obtained from Affymetrix 430A and 430B microarrays, which provide more than 45,000 probe sets, almost all of which are represented by 11 PM probes and 11 MM probes.

### Microarray data reduction

In addition to the HWT1 method, microarray data were processed with Microarray Suite 5.0 (MAS5) software [31,32], RMA [20], PDNN [17] and dChip [15].

### HWT1 weighting

Individual probe intensities from Affymetrix U74Av2 microarrays were log_2_-transformed and normalized to a standard array-wide mean and standard deviation. For each probe, mean squared deviations within strains (MS_w_) and between strains (MS_b_) were calculated by analysis of variance of the log-transformed, normalized expression. In the interests of speed, age and sex of animals were not included as cofactors in the analysis of variance. Raw heritability was estimated as (MS_b _- MS_w_)/(*n*MS_t_), where *n *is the average number of replicates per strain and MS_t _is total variance (excluding strains without replicates, if any) [33]. Adjusted heritability was derived from raw heritability by assigning values of 0 and 1, respectively, to raw heritability values below 0.0 or above 1.0. Weights for each probe were calculated by dividing the adjusted heritability by the mean adjusted heritability for all probes in the probe set. Finally, expression estimates for each probe set and strain were calculated by an unweighted average of replicates within each strain and a weighted average of those probe-specific means, using the weights just described. To avoid division by zero, and to avoid using weights based on very small heritabilities, probes in a probe set were assigned a weight of 1.0 if the average adjusted heritability of those probes was less than 0.01. That is, expression for those probe sets was calculated from an unweighted average. The number of probe sets affected by this treatment was 5 (0.04%), 33 (.26%) and 4,178 (9.3%), respectively, for the Brn, HSC and Cer datasets. The large number of affected probe sets for cerebellum is consistent with the high number of negative raw heritability estimates for this dataset.

As explained under Results, polymorphisms between C57BL/6J and DBA/2J in probe target sequences would be expected to affect hybridization of Affymetrix probes, generating an apparent QTL mapping to the location of the transcript. To reduce the effect of this type of artifact, we prepared, from sequence information for the two strains, a list of 614 probes having polymorphisms in target sequences of probes on the U74Av2 microarray. During the weighting procedure described above, these probes were assigned a weight of 0, removing their contribution from any QTL for their probe set. This procedure was not applied to the cerebellum data, which came from a different microarray.

Among the HSC data, a systematic difference between the first and second batches described above would have greatly inflated all heritability estimates. To avoid this problem, heritability estimates were based on the first batch only, but all data were weighted and used for QTL mapping. Among cerebellum data, weighting was necessarily based only on replicated samples, most of which consisted of one sample from each batch. Any systematic batch difference would decrease heritability estimates. As with HSC data, cerebellum data from all strains was included in QTL mapping, weighted according to heritability estimates based on the strains with replicated samples.

### QTL mapping

Heritability-weighted averages were evaluated by regression against marker genotypes, where alleles at markers were coded as -1 or 1. In the interest of speed, regression was performed only at marker locations, but the limitations of this restriction were minimized by using 779 markers (described as the BXD genotype set at WebQTL [10]). Although WebQTL includes values for parental lines and F1 related to the BXD RI lines, these were not used in QTL mapping [26]. For each microarray trait value, the locus yielding the maximum LRS [3] and the LRS itself were retained. An empirical P-value was then calculated for this LRS by a permutation test [34]. Microarray trait values were permuted randomly among the progeny individuals 1,000 times and the regression analysis is repeated for each permuted dataset. If the original LRS fell within the distribution so that at least 10 values from permuted sets were greater, a P-value was calculated from the rank of the original LRS in the distribution. If a P-value could not be calculated, additional permutations are performed, until a P-value could be calculated or until 1,000,000 permutations had been performed. For each microarray trait, four data values were retained, the locus yielding the highest LRS, the LRS and regression coefficient at that locus, and the P-value of the LRS. To evaluate significance, all results from one microarray experiment were sorted by P-value, and the significance of the smallest P-values was determined by the method of Benjamini and Hochberg [25], using a false-discovery rate of 20%.

Mapping was performed with custom software, called QTL Reaper, written in Python and C for Linux. This software will be described fully in a subsequent publication but is currently available from SourceForge [35]. Calculations were performed on an eight-node Linux cluster, which achieved processing rates of about 5,000 genome scans per cpu-second. Most processing time was spent on the small fraction of probe sets requiring more than 10^5 ^permutations.

### Effective number of probes

Within a probe set, the weight of each probe may vary from 0 to the number of probes in the set, *n*. The effective number of probes *f *in a weighted average is defined as



where *w*_*i *_is the weight of probe *i*. This index varies from 1 to *n*. It is equal to *k *if *k *of the probes are weighted equally, and it is less than *k *if *k *of the probes are weighted unequally (with zero weight for the *n *- *k *remaining probes).

### Data availability

The HSC dataset has been placed in GEO. The accession number is GSE2031, and the arrays are GSM36673 to GSM36716. The Brn and Cer datasets are now both accessible from WebQTL [13].
